# Reticulocyte-prone malaria parasites predominantly invade CD71^hi^ immature cells: implications for the development of an in vitro culture for Plasmodium vivax

**DOI:** 10.1186/1475-2875-12-434

**Published:** 2013-12-01

**Authors:** Lorena Martín-Jaular, Aleix Elizalde-Torrent, Richard Thomson-Luque, Mireia Ferrer, Jose C Segovia, Esperanza Herreros-Aviles, Carmen Fernández-Becerra, Hernando A del Portillo

**Affiliations:** 1 ICREA at Barcelona Centre for International H ealth Research (CRESIB, Hospital Clínic-Universitat de Barcelona)/ISGlobal, Barcelona, Spain; 2 Cell Differentiation and Cytometry Unit, Division of Hematopoietic Innovative Therapies, Centro de Investigaciones Energéticas Medioambientales y Tecnológicas and Centro de Investigación Biomédica en Red de Enfermedades Raras (CIEMAT/CIBERER), Madrid, Spain; 3 Tres Cantos Medicines Development Campus, GlaxoSmithKline, Tres Cantos, Spain; 4 Present address: Centre d’Etudes d’Agents Pathogènes et Biotechnologies pour la Santé, Montpellier, France

**Keywords:** Reticulocytes, Plasmodium yoelii, Plasmodium vivax, In vitro culture, CD71 cells

## Abstract

**Background:**

The lack of a continuous i*n vitro* culture system for blood stages of malarial parasites with a unique tropism for reticulocytes, such as *Plasmodium vivax* and the *Plasmodium yoelii* 17X reticulocyte-prone strain, hinders research in these organisms. The maturation of reticulocytes into erythrocytes is a complex process involving the selective removal of membrane proteins such as the transferrin receptor, CD71. In order to advance in the characterization of infected cells during experimental infections of BALB/c mice with *P. yoelii* 17X, CD71 expression in erythroid cells (TER119^+^) was assessed and *in vitro* culture of *P. yoelii* 17X was attempted by adding reticulocytes highly expressing CD71.

**Methods:**

BALB/c mice were infected with *P. yoelii* 17X-GFP transgenic parasites and erythroid cells (TER119^+^) were analysed in blood, spleen and bone marrow cells. TER119, CD71 and GFP expression was assessed at different points post-infection by flow cytometry. Moreover, *in vitro* culture of *P. yoelli* 17X was attempted by adding red blood cells (RBCs) from mice with a pyruvate kinase deficiency, which contain high percentages of CD71^hi^ cells in peripheral blood as compared to healthy animals.

**Results:**

A predominance of erythroid cells lacking expression of CD71 (CD71^-^) was observed in peripheral blood and spleen in normal and infected animals up to ten days post-infection (pi). At ten days pi, however, a dramatic temporal switch to erythroid cells highly expressing CD71 (CD71^hi^) was observed in the spleen and at day 15 pi in peripheral blood of the infected cells. A distribution of erythroid cells expressing differently CD71 was noticed in the bone marrow. Yet, similar to peripheral blood and spleen, a predominance of CD71^hi^ cells was observed at 15 days pi. Remarkably, CD71^hi^ cells were the cells predominantly infected in these organs as well as in peripheral blood. Attempts were thus made to culture *in vitro* the *P. yoelli* 17X strain by adding RBCs from pyruvate kinase-deficient mice containing high percentages of CD71^hi^ cells in peripheral blood.

**Conclusions:**

The parasite preference for immature cells that are rare in normal peripheral blood could have important implications for the development of an *in vitro* culture system for *P. vivax*.

## Background

Reticulocytes represent the invasion target for a number of malarial parasites, including *Plasmodium vivax* and the rodent malaria *Plasmodium yoelii* 17X strain [1,2]. Arguably, it is thought that this tropism has hindered the development of a continuous *in vitro* culture system for blood stages of these species. Yet, several attempts to establish the *in vitro* culture system for blood stages of *P. vivax* by adding different sources of reticulocytes [3] have been unsuccessfully tried over the past 100 years.

Enucleation of erythroblasts in the bone marrow is the source point of reticulocytes that after a brief period of time in the erythropoietic tissue are released into circulation where they mature into erythrocytes. Interestingly, it has been long established that reticulocytes are a heterogeneous cell population composed of cells in different stages of differentiation [4-6]. The transformative process of maturation of reticulocytes includes loss of organelles through apoptosis and autophagy [7], and the remodelling of the plasma membrane through the selective removal of proteins, notoriously the transferrin receptor CD71 in nanovesicles termed exosomes [8].

Here, to better characterize the cells infected during *P. yoelii* 17X-BALB/c experimental infections, CD71 expression in parasitized erythroid cells (TER119^+^) from peripheral blood and erythropoietic organs have been assessed. TER119 is a marker for erythroid cells from the early pro-erythroblast to mature erythrocyte stages of development [9] and CD71 is the transferrin receptor known to be released in exosomes during erythrocyte maturation [8], The results suggest that the more immature reticulocytes highly expressing CD71 are the predominant target cell for invasions. As a proof of concept, the *in vitro* culture of the *P. yoelii* 17X strain was tried by adding red blood cells (RBCs) from mice with a pyruvate kinase deficiency (PKD) containing higher percentages of CD71^hi^ cells in peripheral blood than healthy animals [10].

## Methods

### Mice

All the animal studies were performed at the animal facilities of Hospital Clinic in Barcelona in accordance with guidelines and protocols approved by the Ethics Committee for Animal Experimentation of the University of Barcelona CEEA-UB (No 87/12). Female BALB/c mice of seven to nine weeks old were obtained from Charles River Laboratories. AcB55 (C57Bl/6 × A/J) mice (also recorded as PKD mice) carrying a point mutation at nucleotide 269 (T → A) of the *pklr* gene [10] were obtained from Emerillon Therapeutics (Montreal, Quebec, Canada) and bred at the animal house of CIEMAT (registration number 28079-21A). When needed, eight to ten-week old PKD mice were shipped to Barcelona and after an eight-day housing period, mice were used as reticulocyte donors for *in vitro* culture experiments.

### Parasites

*Plasmodium yoelii*-GFP transgenic lines were generated as described before [11]. To generate *P. yoelii* 17X parasites transgenic for the red fluorescent protein mCherry, schizonts of *P. yoelii 17X* were transfected with the pL0017-mCherry plasmid (kindly donated by V T Heussler, University of Bern, Switzerland) following standard methodologies [12].

### Flow cytometry analysis

Blood, spleens and bone marrow were obtained from control and infected mice on days 1, 3, 5, 10, 15 and 30 post-infection (pi) and single-cell suspensions were prepared. Blood was obtained by intracardiac puncture. Spleens were homogenized and passed through a nylon mesh of 70 μm (352350, BD) to create a single-cell suspension. Bone marrow cells were collected by flushing the femurs and tibias with RPMI medium. The cells were counted by an automatic counter (Countess™, Invitrogen™) and 5 × 10^6^ cells were used for the staining. Before addition of specific fluorescently labelled antibodies, the cells were pre-incubated with Seroblock anti-Fc receptor antibody (BUF041A, AbDSerotec) for 10 min. Cells were stained with PerCP anti-TER119 (116225, Biolegend), PE conjugated anti-CD71 (ab25622, Abcam) at 4°C for 30 min. Samples were acquired on a BD LSRFortessa Flow Cytometer and analysed with FlowJo software. Non-nucleated erythroid cells were defined according SSC/FSC profile and TER119 expression. TER119^+^ cells were analysed for CD71 and GFP expression. The percentage of different populations of cells was calculated as a function of erythroid cells. Data are representative of 200,000 events collected. Results are expressed as the mean ± SEM and were analysed using GraphPad Prism software.

### Electron microscopy analysis

Blood cells from *P. yoelii* 17X infected BALB/c mice at day 15 pi were stained with CD71 antibody (ab25622, Abcam) as described above. CD71^hi^ cells were sorted using a BD Aria II. For ultrastructural analysis, cells were fixed in 2% paraformaldehyde and treated with Karnovsky’s fixative. Small pieces of 1 sq mm were fixed in the same fixative at 4°C for at least 24 hours, post-fixed in 1% osmium tetroxide and dehydrated in acetones before embedding in Spurr resin. Ultrathin (70–90 nm) sections of the spleen were obtained on an ultramicrotom Ultracut E (Reichert-Jung) equipped with a diamond knife (Diatome). Ultrathin sections were stained with 2% uranyl acetate and lead citrate [13] to allow high-contrast imaging. Image acquisition was performed using a JEOL 1010 TEM operating at 80 kV with a Bioscan 792 camera (Gatan MultiScan cameras).

### In vitro culture

Blood from PKD and infected mice was obtained by intracardiac puncture and passed through a hand-made CF11 cellulose filter to remove the leucocyte population [14]. Non-synchronized infected blood was mixed with blood from PKD mice in order to obtain parasitaemia between 0.25 and 4% and resuspended at a density of 1.5×10^7^ cells/ml in McCoy medium containing 10% FBS and penicillin/streptomycin. Two-hundred μl of cells prepared as described above were cultured in 96-well plates at 37°C and with 5% C0_2_, 5% 0_2_ and 90% nitrogen. Cells from PKD mice were maintained at 4°C with no major variations in the percentages of reticulocytes (around 45%). 0.5×10^5^ cells from PDK mice maintained at 4°C were added to each well every day. For examination of cultures, the entire content of each well was drawn off and cells counts were monitored by Giemsa staining and flow cytometry. In some experiments, blood from PKD mice was stained with the viable dye CFSE [15] and used in experiments with mCherry transgenic parasites in the same conditions as described above. Briefly, CFSE Vybrant CFDA-SE Cell Tracer Kit (C34554, Invitrogen) was used to label cells according to the manufacturer’s instructions. Cells were suspended in 5 μM CFSE solution in PBS for six minutes at room temperature. The reaction was stopped by dilution and cells were then washed in PBS. Parasitaemia was assessed on a BD LSRFortessa Flow Cytometer and analysed with FlowJo software.

## Results

**Figure 1:**
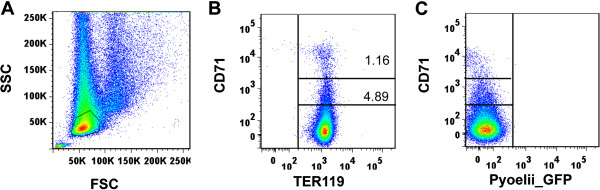
**Gating strategy used for the flow cytometry analysis.** Blood cells from non-infected mice were labelled with CD71 and TER119 specific antibodies and analysed for GFP transgenic parasites in a BD LSRFortessa Flow Cytometer. **(A)** The gate was set to exclude debris and include non-nucleated erythroid cells. **(B)** Numbers in the upper-right insets show percentages of TER119^+^CD71^+^ stained reticulocytes. TER119^+^CD71^+^ cells were gated into two populations according to differences in CD71 intensity. **(C)** TER119^+^ cells from infected mice were analysed according CD71 and GFP (parasite) expression. Data are represented as mean ± SEM of three mice per group.

**Figure 2:**
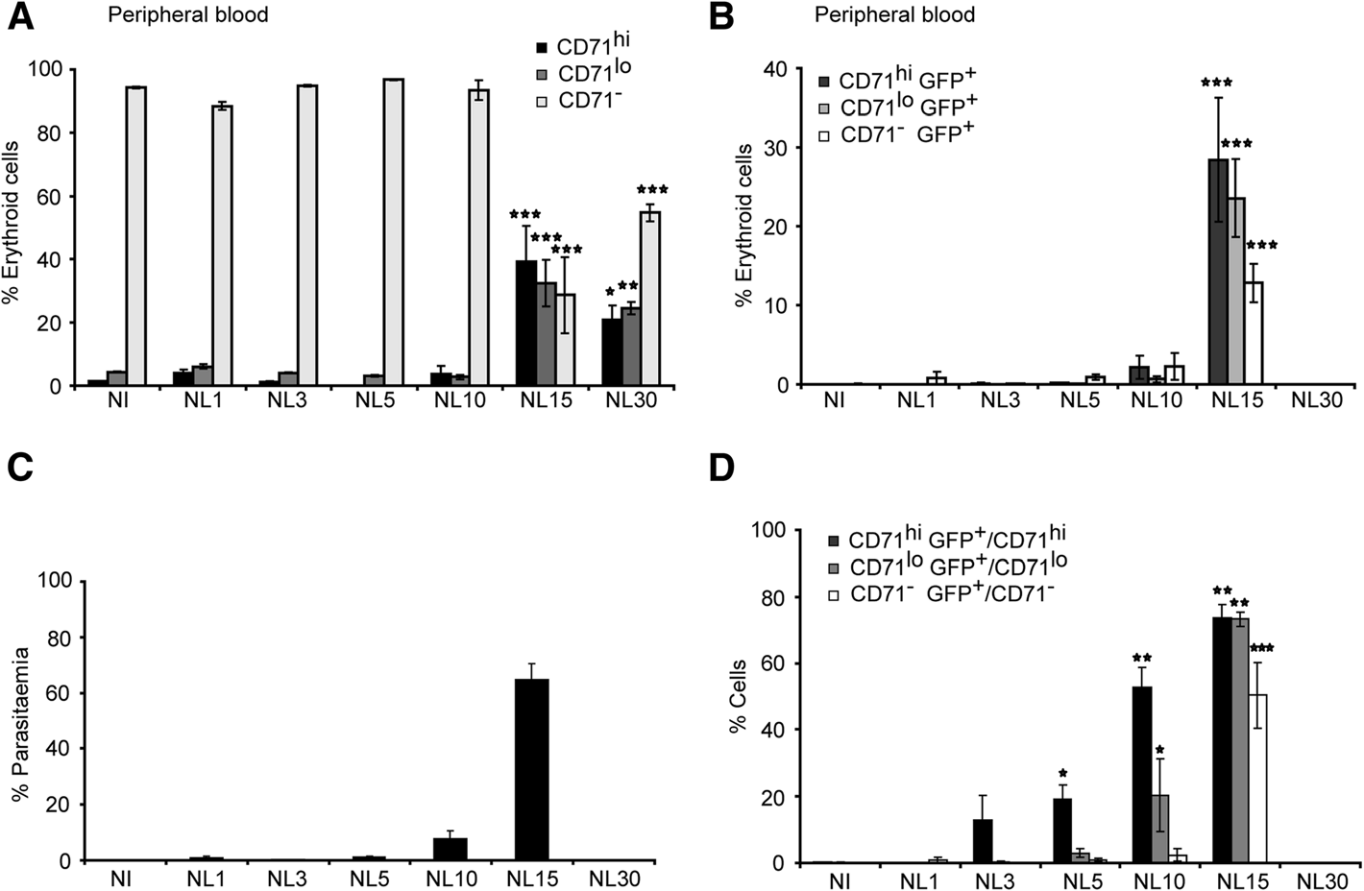
**Flow cytometric analysis of cells from peripheral blood on different days post-infection.** BALB/c mice were infected ip with 10^5^*P. yoelii* 17X-GFP parasites and peripheral blood was analysed at different days post-infection. Cells were labelled with CD71 and TER119 specific antibodies and analysed for GFP transgenic parasites in a BD LSRFortessa Flow Cytometer. The gate was set to exclude debris and include erythroid cells. TER119^+^CD71^+^ cells were gated into two populations according to differences in CD71 intensity. **(A)** Percentages of CD71^hi^, CD71^lo^ and CD71^-^ as a function of erythroid cells from blood are expressed as the mean ± SEM of three mice. **(B)** Percentages of infected cells (CD71^hi^GFP^+^, CD71^lo^GFP^+^ and CD71^-^GFP^+^) as a function of erythroid cells from blood are expressed as the mean ± SEM of three mice. **(C)** Parasitaemias and **(D)** percentages of infected cells (CD71^hi^GFP^+^, CD71^lo^GFP^+^ and CD71^-^GFP^+^) as a function of CD71^hi^, CD71^lo^ and CD71^-^ respectively are expressed as the mean ± SEM of three mice. Data were evaluated by analysis of variance for each cell type in the different time points post-infection and *versus* non-infected (*P <0.05, **P <0.01 and ***P <0.001) (Dunnet post hoc test *versus* NI). Non-infected animals (NI); animals infected with the *P. yoelii* 17X nonlethal-GFP strain (NL).

The *P. yoelii* 17X non-lethal strain has a preferential tropism for reticulocytes [2]. Reticulocytes are precursors of erythrocytes that present different stages of differentiation [4-6]. Therefore, advantage was taken of a *P. yoelii* 17X transgenic line constitutively expressing GFP throughout the asexual blood stages [11] to determine if there was predominant invasion in any one particular stage of reticulocyte maturation. To do so, *P. yoelii* 17X-GFP infection of BALB/c mice for up to 30 days using TER119 and CD71 as “surrogate surface markers” of reticulocyte differentiation was assessed by flow cytometry. The percentages of different populations of cells were calculated as a function of erythroid gated cells (TER119^+^) (Figure 1). As expected, mature erythrocytes mostly lacking expression of CD71 (CD71^-^) were the most abundant erythroid cell population (around 95%) in peripheral blood of uninfected mice (Figure 2A). In infected mice, however, a significant decrease in the numbers of CD71^-^ with a parallel increase in more immature cells (CD71^lo^ and CD71^hi^), was observed after 15 days pi (Figure 2A). Moreover, GFP parasites were found in different CD71 cells with a predominance of infection in CD71^hi^ cells at day 15 pi (Figure 2B). As shown by a previous study (10), infected mice cleared the blood stage parasite infection at approximately day 20 p.i. (Figure 2C). It is interesting to note that predominant and significant infection of CD71^hi^ cells in peripheral blood was demonstrated during the first days pi whereas at the peak of parasitemias infection was observed in all CD71 cells (Figure 2D).

**Figure 3:**
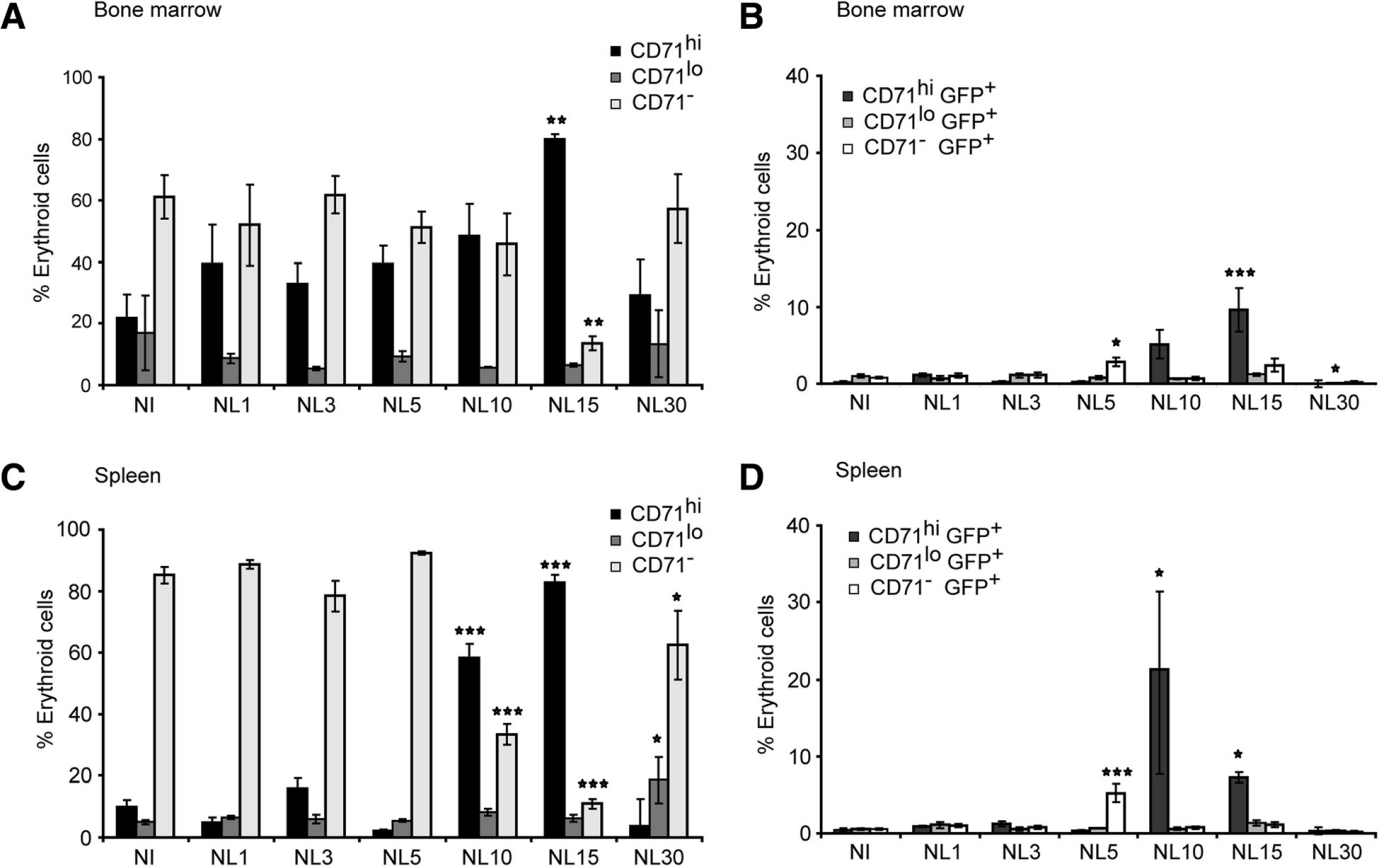
**Flow cytometric analysis of cells from erythopoietic organs on different days post-infection.** BALB/c mice were infected ip with 10^5^*P. yoelii* 17X-GFP parasites and cells from bone marrow and spleen were analysed at different days post-infection. Cells were labelled with CD71 and TER119 specific antibodies and analysed for GFP transgenic parasites in a BD LSRFortessa Flow Cytometer. The gate was set to exclude debris and include non-nucleated erythroid cells. TER119^+^CD71^+^ cells were gated into two populations according to differences in CD71 intensity. Analyses of bone marrow **(A, B)** and spleen **(C, D)** erythroid cells. **(A, C)** Percentages of CD71^hi^, CD71^lo^ and CD71^-^ as a function of erythroid cells are expressed as the mean ± SEM of three mice **(B, D)**. Percentages of infected cells (CD71^hi^GFP^+^, CD71^lo^GFP^+^ and CD71^-^GFP^+^) as a function of erythroid cells are expressed as the mean ± SEM of three mice. Data were evaluated by analysis of variance for each cell type in the different time points post-infection and *versus* non-infected (*P <0.05, **P <0.01 and ***P <0.001) (Dunnet post hoc test *versus* NI). Non-infected animals (NI); animals infected with the *P. yoelii* 17X nonlethal-GFP strain (NL).

Previous studies in this rodent malaria model demonstrated that upon infection there was induction of reticulocytaemia and spleen erythropoiesis [11,16]. Therefore, to evaluate whether the observed increase in immature CD71^hi^/CD71^lo^ cells in peripheral blood during experimental infections parallel changes in erythropoietic organs, animals were infected with 10^5^*P. yoelii* 17X- GFP parasites and populations of erythropoietic cells in the bone marrow and the spleen were investigated. Immature cells (CD71^hi^ and CD71^lo^) represented around 40% of erythroid cells in bone marrow of non-infected mice and not significantly different cell profiles were observed upon infections until day 15 pi (Figure 3A). At this day pi, however, there was a temporal switch with CD71^hi^ cells reaching 80% and being the cells mostly infected (Figure 3A-B). In spleens of non-infected mice and of infected mice, immature CD71^hi^ and CD71^lo^ cells represented around 15% of erythroid cells up to 10 days pi (Figure 3C). On day 10 pi however, a highly significant temporal switch with a predominance of CD71^hi^ cells and CD71^hi^-infected cells was observed (Figure 3C-D). Altogether, these data strongly demonstrate that the *P. yoelii* 17X reticulocyte-prone non-lethal strain predominantly invades CD71^hi^ cells.

**Figure 4:**
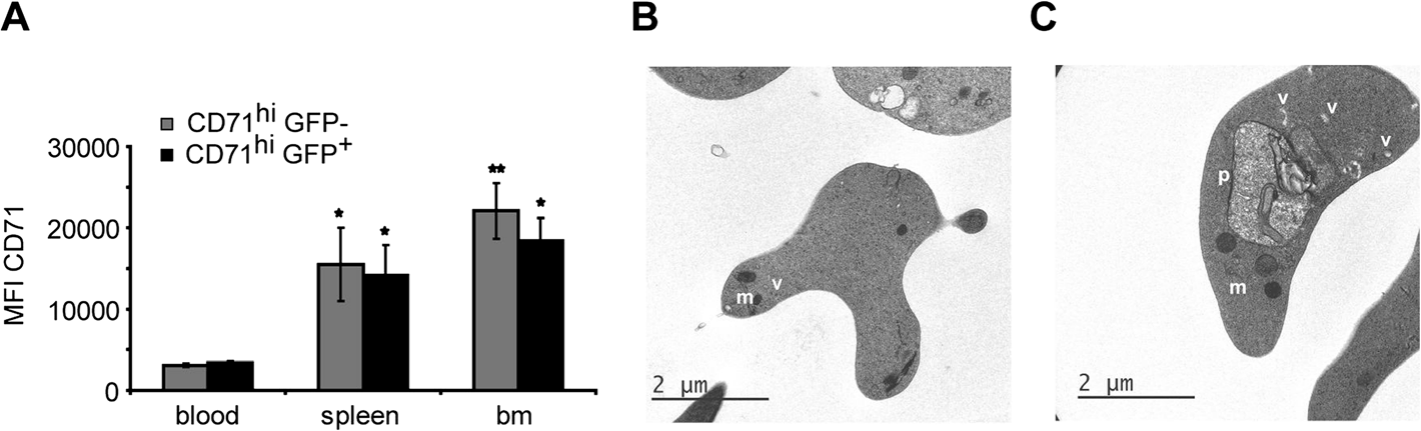
**Analysis of erythroid cells at day 15 pi.** Blood, spleen and bone marrow cells from mice infected with the GFP transgenic parasites were stained with specific antibodies. **(A)** The mean fluorescence intensity (MFI) of CD71^hi^ cells was measured in the erythroid population at day 15 pi. Results represent the mean ± SEM of two independent experiments with three mice each. Data were evaluated by analysis of variance for each cell type separately and *versus* blood (*P <0.05 and **P <0.01) (Dunnet post hoc test). Electron microscopy of non-infected **(B)** and infected **(C)** blood CD71^hi^ cells obtained after sorting showed early reticulocytes containing different organelles. Mitochondria (m), vesicles (v) and parasite (p).

Levels of CD71 expression in the more immature cells from blood, spleen and bone marrow at day 15 pi were next examined. The mean fluorescence intensity (MFI) of CD71 was higher in bone marrow and spleen than in peripheral blood (Figure 4A). This fact, together with the presence of high percentages of infected CD71^hi^ GFP^+^ cells in erythropoietic tissues and both CD71^hi^GFP^+^ and CD71^lo^GFP^+^ in peripheral blood before the peak of parasitemia strongly indicate that parasites initiate infections by predominantly invading CD71^hi^ cells in erythropoietic organs. Indeed, uninfected (Figure 4B) and infected (Figure 4C) reticulocytes appear in blood as cells containing a multitude of organelles only seen in immature reticulocytes.

**Figure 5:**
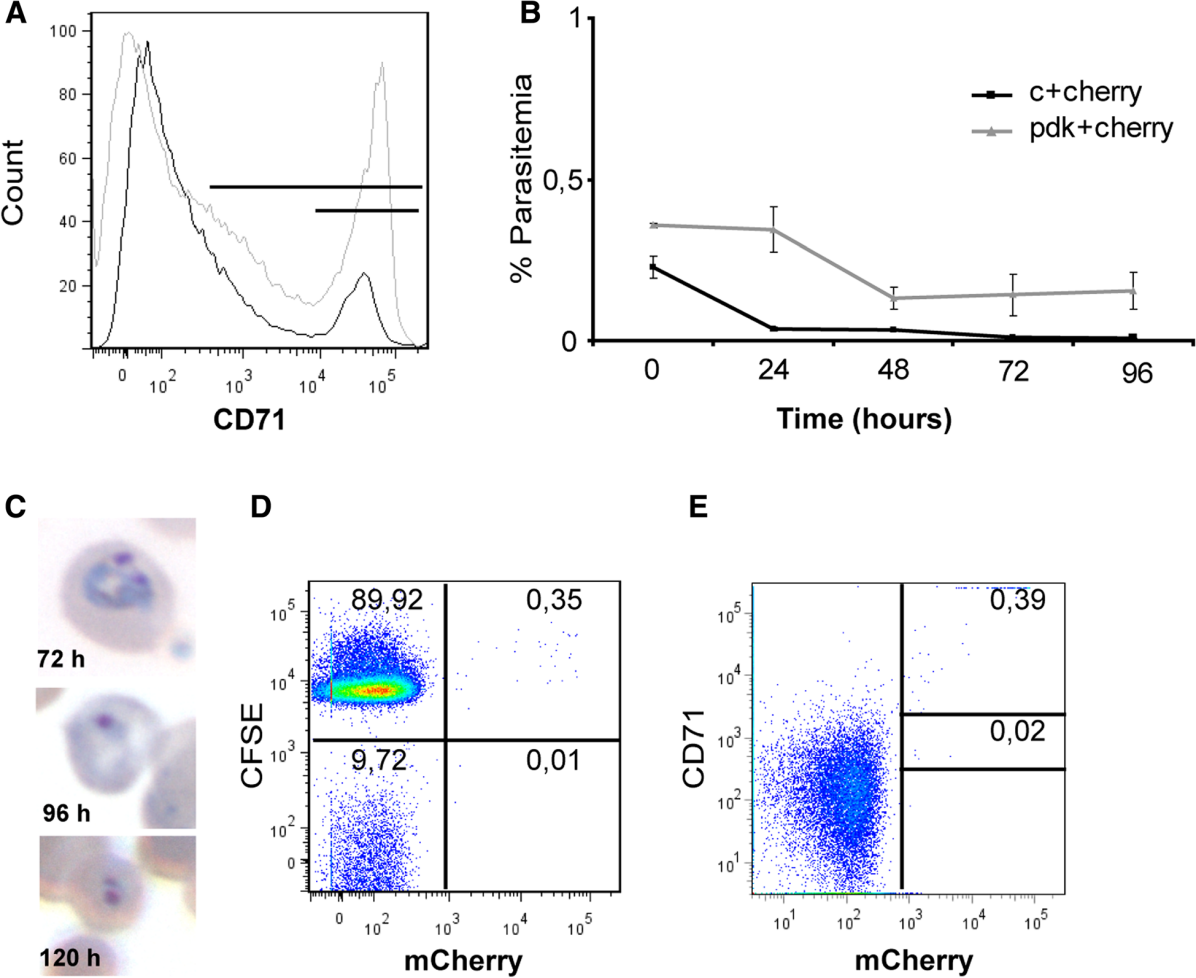
**CD71**^**hi **^**immature cells as a target for the development of the *Plasmodium yoelii *17X-mCherry strain *in vitro *culture. (A)** CD71 expression of blood cells from wild type mouse (black line) and a PKD mouse (grey line). Results were representative of three independent experiments. Percentages of CD71^+^ cells were 47,82% for PKD mice and 6,89% in control mice. CD71^hi^ cells represent 24,40% for PKD mice and 4,92% for control mice. **(B)** Percentages of parasitaemia during *in vitro* culture of *P. yoelii* using PKD reticulocytes. Results were representative of four independent experiments. **(C)** Giemsa staining images of blood smears after 72, 96 and 120 hours of *in vitro* culture. **(D)** Representative dot plot of CFSE-PKD/mCherry-parasite signal for erythroid cells after 72 hours of *in vitro* culture. **(E)** Representative dot plot of CD71-PE/mCherry-parasite signal for erythroid cells after 72 hours of *in vitro* culture.

The predominant invasion of CD71^hi^ cells that are rare in normal peripheral circulation could have implications for the *in vitro* culture of reticulocyte-prone malarial parasites. To address this possibility, attempts were made to culture *in vitro* blood stages of the *P. yoelii* 17X strain using reticulocytes from a rodent model deficient in pyruvate kinase which contains high levels of CD71^hi^ cells compared to normal mice [10] (Figure 5A). Culture of *P. yoelii* parasites with reticulocytes from PKD mice resulted in sustained parasitaemia for 120 hours (Figure 5B). Moreover, Giemsa-staining revealed the presence of viable ring stages at 72, 96 and 120 hours, strongly indicating re-invasion (Figure 5C). To unequivocally demonstrate invasions into reticulocytes from PKD mice, cells from PKD mice were stained with CFSE, co-cultured with *P. yoelii*- mCherry parasites and analysed by flow cytometry. After 72 hours of culture, all parasites were observed inside CFSE^+^ cells (Figure 5D) and CD71^hi^ cells (Figure 5E).

## Conclusion

These data strongly indicate that immature reticulocytes highly expressing CD71 (CD71^hi^), rarely observed in peripheral blood circulation in normal BALB/c mice, are the cells predominantly invaded by the reticulocyte-prone *P. yoelii* 17X strain. In humans, these cells probably represent R1 reticulocytes formed immediately after enucleation in the marrow as opposed to mature R2 reticulocytes released into the peripheral blood circulation [6]. Blood R2 reticulocytes could be in a late maturation process with advanced autophagic events that impairs parasite survival [7]. The use of immature CD71^hi^ reticulocytes obtained from the bone marrow and/or from patients with different haemolytic anaemias containing high percentages of immature CD71^hi^ reticulocytes is guaranteed to try advancing the development of a continuous *in vitro* culture system of blood stages for *P. vivax*.

## Competing interests

The authors declare that they have no competing interests.

## Authors’ contributions

LMJ, JCS, CFB and HAP designed the experiments; LMJ, AE, RTL and MF performed the experiments; LMJ and AE analysed the data; LMJ and HAP wrote the manuscript. EH significantly contributed to the completion of this project. All authors read and approved the final manuscript.
